# Experiences of Austrian mothers with mobility or sensory impairments during pregnancy, childbirth and the puerperium: a qualitative study

**DOI:** 10.1186/s12884-017-1388-3

**Published:** 2017-06-26

**Authors:** Barbara Schildberger, Christoph Zenzmaier, Martina König-Bachmann

**Affiliations:** 1 University of Applied Sciences for Health Professions Upper Austria, Linz, Austria; 2 University of Applied Sciences Tyrol, Innrain 98, 6020 Innsbruck, Austria

**Keywords:** Birth, Disability, Maternity care, Perinatal care, Pregnancy

## Abstract

**Background:**

Approximately 8% of all women of childbearing age in Austria live with permanent impairments. In everyday life, women with disabilities face various challenges and discrimination, among which the issue of pregnancy and motherhood, in particular, is often considered taboo, and their parenting abilities are doubted. Knowledge in the medical field about the experiences of women with disabilities during pregnancy, childbirth and the puerperium is limited.

**Methods:**

To investigate the personal meanings and experiences of women with disabilities in regard to pregnancy, childbirth and the puerperium, in-depth individual, semi-structured interviews were conducted with ten mothers with various mobility or sensory impairments who reside in Austria. The qualitative interview data were analyzed using the qualitative content analysis proposed by Mayring.

**Results:**

Three main themes or categories emerged from the inductive content analysis, namely, (i) the social network, (ii) self-efficacy and self-awareness and (iii) communication, transparency and information. Participants reported limited acceptance of their life decisions and experienced an environment of discriminatory attitudes. They experienced a lack of support and lack of confidence in their parenting abilities, which negatively influenced their self-efficacy and self-awareness. Violations of personal borders and a feeling of being watched and controlled were reported. Communication with health care professionals was often characterized by mutual aspects of fear, uncertainty and awkwardness, as perceived by women with disabilities. Adequate information about pregnancy, childbirth and the puerperium, particularly about measures taken and interventions applied, was frequently missing.

**Conclusion:**

Heath care facilities need to be structured to ensure ease of access for women with disabilities. Education should be offered to health care professionals to improve knowledge about care for women with disabilities and to strengthen communication skills. All necessary information needs to be prepared and provided in an adequate manner. The establishment of a health-promoting environment for mothers, their children and their families requires a sensitive, respectful and non-judgmental attitude of society toward women with disabilities during pregnancy, childbirth and the puerperium.

**Electronic supplementary material:**

The online version of this article (doi:10.1186/s12884-017-1388-3) contains supplementary material, which is available to authorized users.

## Background

The global prevalence of disability, defined as experiencing significant functioning difficulties in everyday life as an adult, is estimated at 15.6%. In higher-income countries, the prevalence rate of disability in the age group of 18 – 49 years is 6.4% [1]. Of women of childbearing age (15 – 44 years) in Austria, approximately 8.1% live with permanent impairments [2]. In everyday life, women with disabilities face different challenges and aspects of discrimination, such as a lack of barrier-free facilities or social exclusion from mainstream society, including employment options [3]. The issue of pregnancy and motherhood for disabled women is still taboo or viewed critically, and their parenting ability is often brought into question [4, 5].

Worldwide, the prevalence of disability among childbearing women varies greatly, depending on the country and definition of disability. In Austria, the birth rate of women with disabilities is not recorded in national birth statistics. In the UK, a large-scale, population-based study of women who had recently given birth found a prevalence of chronically limiting conditions or disabilities of 9.4% [6]. Among the respondents in the 2007–2008 Massachusetts Pregnancy Risk Assessment Monitoring System, 4.9% reported having a disability [7]. Women with disabilities were more likely to use social health care programs and to have emergency room visits, hospital admissions during pregnancy, cesarean deliveries, preterm deliveries and low-birth-weight infants, and they were equally or less likely to receive adequate prenatal care compared with women without disabilities [8]. Lawler et al. conclude that access to service provisions in prenatal care for women with disabilities must be improved. Essentially, buildings must be adapted to provide sufficient accessibility, prenatal classes with sensibilities for pregnant women with disabilities must be provided, professional development for medical experts must be advanced, and sensitization vis-a-vis disability and pregnancy should be generated [9].

Several qualitative studies conducted within the last two decades indicated that women with disabilities faced poor access to maternity services, as well as structural barriers and lack of appropriate infrastructure [10–12]. Moreover, women with disabilities reported limited contraceptive options and inadequate sexual health or reproductive services and support. They experienced insensitivity and lack of knowledge about disabilities on the part of health care providers and limited or insufficient information and inadequate or inopportune support from health professionals (physicians, midwives and nurses) and were confronted with assumptions and discriminatory practices [10–12].

Health care professionals acknowledged their own ‘lack of competence, knowledge and skills’ regarding disability and indicated lack of effective communication and deficient resources in a recent qualitative study [13].

Article 23 of the UN Convention on the Rights of Persons with Disabilities states that parties to the convention must commit themselves to ‘take effective and appropriate measures to eliminate discrimination against persons with disabilities in all matters relating to marriage, family, parenthood and relationships, on an equal basis with others‘ [14]. As of July 2015, 156 countries, including all member states of the European Union, have ratified this convention and pledged to provide equal quality of maternity care for women with disabilities. However, it was reported that many obstetric wards in Austria lack structures that ensure accessibility to care, nursing, counseling and support for women with disabilities [15]. The implemented structure of health insurance-covered Mother-Child-Care comprises five medical exams during pregnancy by a licensed gynecologist, one internal examination, laboratory tests and three optional ultrasound examinations; since 2014, optional counseling by a midwife for 1 h between the 18th and the 22nd week of pregnancy has also been covered. Childbirth can occur at a clinic or at home. Postnatal care usually includes either hospital care or nursing care by a midwife at home until the fifth day after birth. Additional options, such as prenatal classes, acupuncture, breastfeeding groups, baby massage, babywearing courses or yoga, are offered by health care professionals or midwives but must be paid for by the women themselves. Accessibility to such options is frequently limited [15]. The present study examined the experiences of women with mobility or sensory impairments with regard to pregnancy, childbirth and the puerperium in Austria through individual oral interviews.

## Methods

In-depth individual, semi-structured interviews with ten mothers with various mobility or sensory impairments who reside in Austria were conducted between September 2013 and March 2015. Women were recruited via national disability organizations or by way of personal contacts. Interviews occurred either at the participant’s home, the office of the respective disability organization, or the office of the researcher. For participation in the study and inclusion in the sample, the criterion of physical disability and/or sensory impairment was stipulated. Consequently, prospective mothers suffering from mental or psychic disabilities were excluded from participation. Moreover, the disability status had to predate pregnancy. The woman’s age was not a criterion for inclusion or exclusion, nor was the age of the child. At the time of the interviews, the age of the women’s children ranged from 1 to 13 years. The duration of the interviews ranged from 50 to 90 min. A sign language interpreter or a close relative assisted the interviews with deaf women.

In preparation for the interviews, the research team developed an interview guide for the conduct of the semi-structured interviews providing structural standards regarding pregnancy, childbirth, the puerperium and daily family routine (see Additional file 1). The interview guide aimed at eliciting not only the most relevant experiences of the participating women with respect to clinical and external care but also their impressions from interrelations with their social environment. Women were asked how many children they had and how they arranged everyday life, particularly whether they received professional or private assistance in terms of housekeeping and childcare. Interviews focused on how the women experienced and perceived pregnancy, how they and their immediate environment reacted when the pregnancy was revealed, how they prepared for childbirth and motherhood, and whether or not they received assistance during pregnancy and from whom. Women were asked what they perceived as helpful with regard to childbirth, what was important to them, who was present during childbirth, whether they considered the support sufficient and adequate and what they perceived as disruptive. In terms of the puerperium, the interviews emphasized how the women experienced and perceived this period and, in particular, whether care and support were adequate and sufficient and whether they received professional or private assistance in terms of housekeeping and childcare.

The interviews were recorded with the permission of the participants, digitally transcribed and assigned pseudonyms. An analysis of qualitative interview data was performed using the qualitative content analysis proposed by Mayring [16, 17], one of the most well-established qualitative methodologies in social research in German-speaking countries. Accordingly, the transcripts were analyzed by the stepwise inductive construction of codes, which were subsequently sorted into main and subcategories. According to the rules of the qualitative content analysis, the categories were developed in close dependence on the original interview material. To guarantee inter-subjective traceability, categories were discussed among the involved researchers and, in case of discrepancies, a consensus was reached.

## Results

Ten women participated in the study, four with a physical disability and six with sensory impairment (four blind and two deaf women). To describe the sample, demographic information was compiled from interview transcripts. Six women were the mother of one child, while four women had two or three children. At the time the interviews occurred, four were single parents, four were married and two were cohabiting. Out of the ten interviewed women with disabilities, nine were living in urban areas and one in a rural area. A list of the study participants and their pseudonyms is provided in Table 1.

**Table 1:** Study participants

Pseudonym	Disability	Number of children	Age of children
1M	Spastic cerebral palsy	1	<5
2M	Paraplegia after accident	1	<5
3M	Paraplegia after accident	1	<5
4M	Paraplegia after accident	1	>10
5S	Blind after accident	1	5 – 10
6S	Blind from disease	3	5 – 10
7S	Blind from birth	1	<5
8S	Blind from birth	3	>10
9S	Deaf from birth	2	<5; 5 – 10
10S	Deaf from birth	2	>10

From the qualitative inductive content analysis of transcripts from the ten interviews conducted, three main themes or categories emerged: (i) the social network, (ii) self-efficacy and self-awareness and (iii) communication, transparency and information.

### The social network

All the interviewed women were continuously integrated into well-functioning family, neighborly or social networks, which usually provided sufficient support in various situations [18, 19]. Nevertheless, the women reported that overall social acceptance of their life decisions and equal treatment in regard to pregnancy, childbirth and motherhood was missing. The multifaceted statements regarding the social network were assigned to three subcategories: (i) motivation, trust and acceptance; (ii) professional support; and (iii) childbearing and parenting skills.

#### Motivation, trust and acceptance

All of the women articulated the desire for normality and to be accepted as both a woman and a mother by society in general and by their social environment. Social acceptance was particularly missing not only during pregnancy and its notification but also later during motherhood.

*“My mother said it word-for-word. Why do you do this to yourself? Yes – instead of yeah great. … […] and, and they all were happy with my sister, and with me – no one. And we were pregnant at the same time, which made it then so noticeable, yes…. I, that was, that really hurts.” 1M.*

*2M, responding to the final question of the interview regarding whether she wanted to add anything: “Yes, and what I would wish for as well is the society’s confidence. That also all of them accept that we want to live just like anyone else. And I … that still disturbs me a lot. Sometimes more, sometimes less, but you perceive statements from all sides.”*

In their social networks, women were confronted with preconceptions that they had embarked upon pregnancy and motherhood unmindfully and recklessly. However, in contrast to these preconceptions, most of the interviewed women stated that the ultimate decision to become pregnant was usually preceded by detailed planning for the expected changes in their everyday life. Being aware of the challenges, the route from desiring to conceiving and all the way through the actual pregnancy was a process and necessitated arrangements that were motivated by various parameters such as partnership, job situation or age.

*1M, referring to the moment when she became aware that she was pregnant: “I really wanted a child and I myself had this goal, until 40 I wanted my first child. … And it immediately worked out and I just got pregnant. … And I was so happy about that.”*

All the women reported great joy and confidence with respect to pregnancy, childbirth and the puerperium but also fear and doubts, which were additionally intensified by their own family and social environment.

*1M, referring to the reaction of her family when she announced her pregnancy: “They [family] were absolutely shocked. … But the worst thing for me was that my family did not share my delight. I knew they would not be happy. I knew that. But then, it does hurt anyway.”*

*2M, explaining how she perceived the reactions of the societal environment to her pregnancy: “And you just do not know what the others think. But you already felt [their] doubts and insecurities. Yes, and that of course is talked about, especially in such a small place.”*

#### Professional support

In the context of the present study, the notion of professional support was divided into two main categories. The first category was institutionalized services, such as health insurance-covered Mother-Child-Care and supplementary offers such as prenatal classes, as detailed in the background section. The second category was direct personal interactions between health professionals and women with disabilities.

In the conducted interviews, women repeatedly complained about receiving little or insufficient support and assistance.

*7S, after being asked if she did receive or would have needed support during pregnancy: “Ah during pregnancy, unfortunately, I had little support, I have to honestly say that I was very much on my own. … And I have actually perceived that as sad. Because somehow I had no people who would do baby shopping with me or maybe just once do prenatal exercises.”*

With regard to professional support during childbirth, the main criticism was staff shortage and, thus, lack of time spent with health professionals.

*“I just think they should not save expenses, so that basically more midwives should be employed and responding to the needs of the pregnant women because midwives are hopelessly overwhelmed with many births; if there were just two midwives, three midwives…” 7S*

Regarding institutionalized services, the interviewed women mainly referred to the scheduled health insurance-covered Mother-Child-Care examinations. Additional opportunities were, in part, not known or could not be used due to limited accessibility.

*2M, responding to the question of how she prepared for childbirth and motherhood: “I searched the internet for various forums and information regarding mothers in a wheelchair, those who went through that, but unfortunately there was really not much available. There is one German homepage that hasn't been updated for years, and directly in Austria, I couldn’t find anything. Yes, there you find no specific information, go-to persons or those concerned.”*

*1M, referring to additional opportunities, such as prenatal classes, acupuncture and yoga: “… there you likely can’t attend, because it’s not barrier-free. Yes, blah-blah. Then, there is definitely no wheelchair-accessible toilet. […] So, I think there is nothing because this is still novel that women with disabilities or with cognitive impairments get pregnant. Overall, the best, bigoted topic. Ah. And if, then, then it gets complicated. Then, you need a wheelchair-accessible restroom and … yes, it gets complicated for them, for us it is always complicated anyway. But now, the non-disabled have to come to grips with a group of persons they don't offer it to.”*

The interviewed women reported that the exchange of experiences with other mothers with disabilities was a valuable source of a broad range of information, including solutions for everyday problems, such as access for mothers with disabilities to children’s facilities. Typically, it requires the woman’s own initiative to request professional support and resources and information to cope with disability limitations. Women perceived the offered professional support structures to be highly limited, but they refrained from requesting special assistance or services so as not to cause inconvenience.

*“It’s more like defending oneself, that one got pregnant at all. Then you do not also want to get special treatment … and yoga during pregnancy – that seems like special treatment to me. It's not for non-disabled women, but for women with disabilities.” 1M.*

#### Childbearing and parenting skills

The interviewed women frequently mentioned society’s lack of confidence in the parenting abilities of mothers with disabilities.

*7S, responding to the question about how her social environment reacted when they were informed of her pregnancy: “So, a little bit of prejudice, a little bit, like yes, what does she, with her visual disability, her practical blindness, need a child for. She can’t handle it anyway. … I also got that kind of reaction. … There were all kinds of reactions.”*

For some women, this lack of confidence on the part of society was very pronounced and manifested itself in rigorous societal control. Women emphasized that they felt constantly observed by others.

*2M, referring to the situation in the puerperal obstetric ward: “And in some cases, the questions of the gynecologists present at the ward rounds were sometimes really discriminatory. […] I should have said, ‘that's too intimate for me’. You are really on self-display, and this is an experience you have to go through.”*

Women with disabilities experienced difficult and emotionally stressful situations when their ability to care for their children was questioned or criticized.

*“Actually, they [the woman’s parents] wanted to receive child custody, so everything was a little bit (…) I am a little bit haggard and under therapeutic treatment. (…) that was really tearing me up inside! My heart in a way…” 5S.*

*“When he [the child] started kindergarten, it was difficult for him. Every child reacts differently. He is a very vivacious child; he tries everything, and in the beginning, he didn’t know about limits and rules. And then, the kindergarten staff asked me if it was due to me sitting in a wheelchair that I couldn’t set enough limits. That was their thought. That my child is like this because I am sitting in a wheelchair. At that time, and also now, that annoys me beyond any measure.” 2M.*

Despite the perceived lack of confidence in their parenting skills, all the interviewed women reported that they had been able to develop a well-functioning relationship with their children, even if they had to rely on professional assistance in childcare. For example, a woman with a mobility impairment receiving personal assistance told that while her child was having fun with the assistants on the playground, the child still urged the mother to join them.

*“And I was so afraid that she [the child] would love the personal assistant more than me. In practice, that is (thank God) not at all the case. Rather, she sometimes has with them… more fun, I don’t know, at the playground than with me. But still, she wants me to accompany her. Although I told her, I can't do this, I can't do that – ‘But I'll show you that, but look’. So, it is not about the participation, but about watching – being present.” 1M.*

### Self-efficacy and self-awareness

A second main theme that emerged from the interviews is the concept of self-efficacy, the awareness of one’s own capabilities to accomplish certain tasks and to obtain projected ends. Confidence in own abilities ultimately influences the actions and behavior of human beings and is as important a personal resource in dealing with everyday tasks for women with disabilities as for everyone else [20, 21].

In contrast to the perceived lack of confidence in their abilities by others, the interviewed women were at first highly confident in their capabilities to manage pregnancy, childbirth and motherhood, particularly if the pregnancy was planned.

*2M, responding to the question about how she experienced pregnancy: “Yes it [the pregnancy] was planned, it was desired. It then also happened very fast, and we were joyful, and I was also sure that I could do it and that it was the right thing. So, I was always confident, and I never had doubts. But from the physical point of view, it was difficult. I had imagined it would be a little bit easier.”*

For some women, the bodily sensitivity that accompanies pregnancy sharpened their perceptions of their own physicality and represented an important resource for early bonding with the unborn child.

*7S, responding to the question about how she experienced pregnancy: “Pregnancy was basically a new experience, also the physical feeling. Because you are feeling very much, and particularly as a practically blind woman, you have to rely more on your feelings than on other things. So, you are not that distracted. And I really felt very, very much at that time. Also, the bond to the child and how he was growing in me. And when he then started to move, these were beautiful moments to me. … It was just something completely new, something very stable, something strong.”*

Doubts and fears about their skills and competencies that originated from the social environment decisively influenced the self-efficacy of these women with disabilities. The perceived evaluation of their existing skills and resources potentially decreased their self-confidence in relation to motherhood.

*“And it is best not to burden anyone because then there is always a: oh, they cannot do it. … and if you cannot do that, then you are, you may not be a mother. … There is always this, this fear in the background. […] And therefore, it is so hard for us to admit that we need something because it always threatens us, this… yes.” 1M.*

In addition, the insecurity and lack of experience of health care professionals with women with disabilities exacerbated the pregnant women’s lack of self-confidence.

*1M, referring to the situation in the hospital: “Fear, that’s the main topic. They are afraid, I am pregnant myself, me myself, I felt bad and I noticed: everybody was afraid.”*

*3M, describing traumatic experiences during childbirth and perceived insecurity on the part of health care professionals: “…they [the physicians at the hospital] always suggested birth with epidural, but naturally. And they also were … one noticed, they were unsure. They did not really have experience with a woman in a wheelchair. […] At 3 a.m., I was having contractions with a 5-minute interval. And then there was no epidural because the anesthetist was not there. And then, the heart rate of the baby declined during contractions. […] I basically was frightened. […] So, fear for myself. But then, when I noticed the decline of the heart rate, of course, fear for the child. I somehow felt relieved when they told me a cesarean would be performed.”*

Occasionally, women with physical disabilities reacted with uncertainty to the physical changes that occurred during pregnancy, childbirth and postpartum, especially if they could not clearly evaluate and understand the occurring symptoms.

*“And these were real cramps. And I could not determine whether that was because of the urinary tract infection. Or if these were Braxton Hicks contractions.” 3M.*

*“I always needed the assurance that everything was fine. I had the feeling that I did not notice everything. Meaning, I do not feel everything, and I wish I would have had an ultrasound unit at home, so that I … would have been able to check by myself.” 2M.*

Health care professionals sometimes handled topics related to prenatal medicine insensitively. For instance, a woman with spastic cerebral palsy reported that, during a routine “Mother-Child-Care” check-up, she was advised to undergo prenatal screening so that abortion could be initiated in case the screening generated evidence of possible disability of the child.

*“Prenatal diagnosis, I am definitely not doing this. It is purest cynicism. Me myself, I am disabled. I am not doing prenatal diagnosis. No way… And the way the child is, that's the way it is, right? And then he [the gynecologist] suggested something different, with the amniotic fluid, somehow piercing it. I'm not doing that, you know?” 1M.*

### Communication, transparency and information

Regarding communication between women with disabilities and health care professionals (mainly doctors, midwives and nursing staff), traces of fear, uncertainty and awkwardness became apparent.

*“And the nurses, they are all kind, very kind, you can’t object to that. But all of them are scared. […] Well, I had to calm everybody, whereas I myself would have needed some support. […] I always had the feeling: They don’t take this s…, they don’t take me seriously, yes.” 1M.*

These uncertainties and fears were frequently based on lack of information and complicated communications and interactions in the care and support of women with disabilities.

*7S, referring to the situation in the labor ward: “One shall have no reservations. Of course there are women who maybe don’t want this. But I think a caressing hand or a little bit of something, everyone would appreciate during birth. Because you are basically already overburdened. … isn’t it? … Because I think one should simply have no reservations. That what I had wanted at the… … and I think if someone would have been more verbally attentive and better coached me, I think that all wouldn’t have been that dramatic.”*

*“Well, I think people need to inform themselves a little bit. Just inform. I cannot lift my feet there. I cannot bend my feet that much. And there is not ‘the’ spasticity, this is maybe also important.” 1M.*

This perceived aggravation of the interaction and communication between women with disabilities and health care professionals often resulted in the women feeling left alone and forsaken.

*7S, referring to the interactions with health care professionals in the labor ward: “And never, ever did someone tell me what he is doing right now, except the anesthetist who did the epidural. […] And somehow, I felt so left alone. I said ‘I am visually impaired, so have… am practically blind’. And there, I just assumed that somebody would maybe tell me what was going to happen.”*

*“That was when I felt let down. Also, in the last days before the birth, I wasn’t able to turn around by myself; then, I also had the feeling that some know the technique, whereas the other ones, they wish not to have anything to do with you because they do not know how to and what to do.” 2M.*

Attentive listening and being asked questions by health care professionals seemed to unburden and support the women and was experienced as very helpful. In contrast, ignorance and neglect of the concerns and needs of women with mobility or sensory impairments could lead to reduced support, as reported by the interviewed women.

*3M, expressing her wish that health care professionals would communicate frankly without timidity: “For me, it is not unpleasant if someone brings up a question. What do you have? This is totally okay … is there anything we can pay attention to? Do you need anything? Can we provide any kind of help? Is there something that can be done?”*

*“I really think that there needs to be more knowledge. Um, because … to somebody who is blind, you need to explain a lot more, to say a lot more or just have to explain what are you doing at the moment, what’s happening in the room. What or how the room is or where you are lying down now, what you are doing.” 7S.*

This was often aggravated by the fact that shortage of time impaired adequate support.

*“I did ask questions, but I rather had the feeling that time was the problem. So, I asked my mother and my friends to share their experiences.” 9S.*

In addition to shortage of time, communication was complicated by further challenges related to sensory impairments, such as the lack of a sign language interpreter, as reported by a deaf woman.

*“Because if she [midwife] talks somehow and you are in pain and you are woozy from anesthesia, a lot is not quite clear. We somehow had the feeling we would have needed an interpreter during the birth or a midwife who just could sign a little bit. It is just difficult for a woman to read lips while she is suffering.” 9S.*

In particular, women with sensory impairments expressed a need for more detailed information on the actual situation and on the required necessary interventions.

*“A hearing person can somehow understand everything that you need, but a deaf woman would also just need this information: what’s actually happening to me? What’s going on now? … I do not even know right now why I had emergency surgery.” 9S.*

Lack of communication, transparency and information resulted in women being exposed to embarrassing or distressing situations. In this context, a woman with a visual disability recalled the uncomfortable feeling of being unnecessarily exposed with spread legs during childbirth.

*5S, referring to the situation in the labor ward: “Of course, it was uncomfortable in the beginning (…) But just the position … the legs. I am usually not very… how do you say…bashful but rather open, but that indeed is rather uncomfortable (…) I did not even know what they saw or did not see or how it even was and so on …”*

## Discussion

### The social network

The identified subcategories for the research chapter Social Network – (i) motivation, trust and acceptance, (ii) professional support and (iii) childbearing and parenting skills – not only hint at the various attitudes, stances, and emotions found among the participants but also underscore the paramount significance of the social factor to the interviewed women with disabilities.

The interviewed women considered it to be of the utmost importance that their desire for a child as well as their motherhood was acknowledged and respected as something normal by their social environment. However, the desire for normalcy is contradicted by the fact that disability is defined as an observable deviation from normality that directly results from disease, trauma or other health condition [22]. In a society, normality or a normal life is often defined as what is considered a matter of course by the majority of its members. This “normal life” includes the naturalness of sexuality and reproduction. According to the WHO’s current bio-psycho-social model of disability, the term “disability” is defined not as a deviation from normality and an attribute of individuals, but rather as a set of difficulties that individuals may experience in interaction with their social and physical environments [23]. In recent years, the sexual independence of people with disabilities has generally been recognized. Nevertheless, despite the recommendation of the United Nations that reproductive health “implies that people are able to have a satisfying, safe sex life and that they have the capability to reproduce and the freedom to decide if, when and how often to do so” [24], the reproductive life of people with disabilities is still treated as taboo.

Discriminatory attitudes in society and the lack of support from family and friends, as reported by the interviewed woman and addressed in the subcategory ‘Childbearing and Parenting Skills’, make pregnancy and childbearing more difficult. Familial and social support, however, would promote the mother’s well-being and health and strengthen her coping resources [25–27]. Adequate support of disabled mothers is therefore an undeniable prerequisite for the establishment of the basic conditions that are critical for children’s positive development, such as bonding, breast-feeding, food preparation and physical care of the child.

While adequate professional support appears to be especially important in this respect, most of the women complained of a lack of support in this area. Since diverse personal, familial and social protection factors promote the healthy development of children and youth, resource-strengthening measures that foster these protection factors must be integrated into any health promotion and prevention programs [28–30].

It is important that women with disabilities are offered support and assistance from health care professionals and from their social network; however, help and support should never be imposed or overbearing [31–33]. If support turns into intrusion or is perceived as such, a feeling of being under constant observation and societal control might develop. As a consequence, the woman might withdraw and reject help and support to avoid excessive interference from her medical and social environments.

### Self-efficacy and self-awareness

Most of the interviewed women testified that, during pregnancy, they felt highly confident in their capabilities to manage childbirth and motherhood. According to the concept of “self-efficacy”, confidence in one’s own abilities positively affects the assessment, motivation and outcome of decisions and activities [9, 34]. A prerequisite for effective self-efficacy is the development of congruent body sensations and adequate body perceptions. Sensitive body sensation, in turn, is the basis for self-confidence and self-responsibility [35, 36]. During pregnancy, the proper perception of the body can also serve as a source of a sense of security, provided that confidence in the abilities of one’s own body is sufficiently high [37, 38].

While the interviewed women reported that the perception of their own physicality was increased during pregnancy, women with physical disabilities, in particular, occasionally felt insecure when physical changes occurred and would have wished for more diagnostic interventions. This insecurity might result in reduced confidence in their own physical functionality, leading to a higher risk of psychogenic obstetric complications. The development of positive body perceptions can be additionally inhibited if the social environment and/or caregivers counteract it. Thus, lack of confidence in their own physical abilities in combination with deficient knowledge and experience in obstetrical care for women with disabilities can eventually lead to insecurity and fear among women with disabilities. Consequently, in such situations, not only diagnostic investigations but also social support and the exchange of experiences among women could be perceived as helpful [39]. Granted adequate support, women with disabilities may experience pregnancy with minimal anxiety regarding physical signs and symptoms. In this respect, applied concepts in medical care during pregnancy, birth and the puerperium should be evaluated and adapted accordingly whenever and wherever necessary [40–43].

Individual concerns for the well-being of the child must be treated with particular sensitivity in prenatal medical care for women with disabilities, since prenatal screening might be perceived as disparaging and thus might impair the self-efficacy and self-awareness of persons with disabilities, as detailed in the excerpt from an interview with a woman with spastic cerebral palsy presented in the results section. In principle, women with disabilities receive the same counseling as all other pregnant women regarding prenatal screening and the possibilities of severe anomalies. However, medical professionals must use special sensitivity and caution when discussing such issues with women who have congenital disabilities themselves, particularly since prenatal diagnosis could be considered a form of discrimination against persons with disabilities [44].

Self-efficacy, as the conviction to be able to have an impact on the environment and to determine one’s own life, is considered one of the strongest protective factors [45]. Having an impact on the social environment requires the capability to communicate with others.

### Communication, transparency and information

If communicative and interactive capabilities are limited, e.g., due to sensory impairments, alternative or extended forms of communication must be available to convey messages, desires, expectations, and feelings. This, in turn, requires specific skills and further training for health care professionals [46]. Both the deaf and visually impaired women in this study complained of a lack of possibilities to adequately express their needs. Moreover, explanations and information about measures and interventions taken during peripartum care were often experienced as insufficient or inadequate.

In regard to the issue of communication between women with disabilities and health care professionals (mainly doctors, midwives and nursing staff), aspects of anxiety, uncertainty and awkwardness became apparent in the conducted interviews. As a result of their insecurity and lack of knowledge, health care professionals might develop protective mechanisms that incidentally can cause deficits in attentiveness and proper nursing care. This underscores the stipulation that successful support needs to be based on an attentive, respectful and dignified attitude, which is shaped and influenced by personal mindsets and values [47].

As part of the human interactions in health care, anxiety can obviously play a role on the part of both health care professionals and patients. The interviewed women consistently reported their own insecurities and fears, which were in part caused by lack of communication and information or resulted from insecurities they perceived from health care professionals. Coping with pain and anxiety during pregnancy, childbirth and the puerperium must be viewed in a multi-factorial context. It is known that there is a discrepancy between women’s needs regarding anxiety management and standard clinical practice [48]. However, continuous and apparent safe support is described as a key element that can lead to increased pain acceptance [48]. Grantly Dick-Read described the elements of fear, tension and pain as a vicious cycle in which every element could be the starting point. A lack of verbal or non-verbal communication enhances this vicious cycle, and loneliness and ignorance in maternity care are considered one of the main causes of increased pain, tension and fear. Providing supportive care appears to disrupt this cycle and reduces stress [49]. Consistently, lack of information and communication resulted in feelings of being left alone among women with disabilities.

The constructivist point of view suggests that communication is a social process in which the involved people inspire each other to construct reality [50]. The mutual stimulus and participation in reality and actuality of each other’s world should be considered very beneficial with regard to caring for women with disabilities. This attitude implies the asking of questions such as What do you need? What is good for you? and How can I strengthen and support you in the best way? Pregnancy, childbirth and the puerperium are primarily intimate experiences and should be regarded as times of “privacy”. Supporting and accompanying persons should be constantly made aware that they are present in an intimate area of women’s lives in those particular periods [51]. Infringing on personal privacy and inattention to a patient’s intimacy can result in increased feelings of shame. From the interview transcripts, it becomes clear that awareness of these aspects is still quite lacking in the support environment of women with disabilities.

### Limitations

The relevance of this study is clearly limited by its small sample size. Furthermore, the possibility cannot be excluded that the interviewed women, in their particular situation as mothers with disabilities, attempted to provide answers that they thought their environment would expect them to give. This is a general problem in interview methodology; however, it might play an especially strong role in the context addressed in this study. The findings of our study are reflective of a particular cultural and temporal context and thus might not be generalizable to other people or other settings, particularly such as other countries with differing maternity services.

## Conclusion

To the best knowledge of the authors, this is the first qualitative study of the experiences of mothers with disabilities during pregnancy, childbirth and the puerperium in Austria. The interviewed women expressed a deep need for normality and acceptance as wives and mothers; however, they perceived a discriminatory environment and lack of confidence in their abilities. Health care facilities show deficiencies and offer limited support to women with disabilities, who often desire adequate information on pregnancy, childbirth and the puerperium. This is partly caused by insecurity and anxiety on the part of health care professionals in the field of obstetric care for women with disabilities. In this setting, the protection of the sphere of privacy is frequently experienced as inadequate. Women with disabilities reported physical, verbal and emotional violations of their personal spheres and often felt watched and controlled. Prenatal diagnosis and treatment are often comprehended as selection tools, leading to increased reflection and evaluation of the pregnant mother’s own disability. However, effective support can strengthen and promote self-efficacy. Heath care facilities need to be structured so that they assure low-threshold, low-barrier access and equitable care for all patients. Adequate education for health care professionals to improve their knowledge about care, communication and interactions with women with disabilities must be provided to properly address the challenges in this particular field of health care. To establish a health-promoting environment for mothers with disabilities and their children and families, society must have a sensitive, respectful and non-judgmental attitude toward women with disabilities, particularly regarding issues of pregnancy, childbirth and the puerperium.

For comparative reasons and further improvement of this particular field of medical care, future research should address the topic of this present study from the perspectives of medical personnel who are involved in obstetrics care and have been exposed to caring for pregnant women and mothers with disabilities.

## Additional file

Additional file 1: Semi-structured Interview Guide. English translation of the interview guide used in the present study. (PDF 233 kb)
