# Supplementary material for: Experiences of Austrian mothers with mobility or sensory impairments during pregnancy, childbirth and the puerperium: a qualitative study
Source: BMC Pregnancy Childbirth. 2017 Jun 26;17:201. doi: 10.1186/s12884-017-1388-3 (PMC5485540; doi:10.1186/s12884-017-1388-3)
Supplement: Additional file 1: — Semi-structured Interview Guide. English translation of the interview guide used in the present study. (PDF 233 kb) [file 12884_2017_1388_MOESM1_ESM.pdf]

Semi-structured Interview Guide:

Initial conversation and general information:

- ✓ Introduce oneself / welcome
- ✓ Thank participant to volunteer for interview
- ✓ Explain intention of interview
- ✓ Information on guaranteed anonymity
- ✓ Information and participant's consent on recording and transcription
- ✓ Information that participant can oppose to answer questions and are allowed to ask questions at any time
- ✓ Information on timeframe of approximately 45 – 60 minutes

1. **You are mother of X children. In what way do you think, your everyday family life differs from that of other families?**
   a. Household help? (Housecleaner, family assistant, relatives, …)
   b. Help / assistance with childcare?

2. **How did you experience pregnancy / pregnancies?**
   a. Getting to know you were pregnant?
   b. Reactions from social environment?
   c. Individual experience?
   d. Support during pregnancy? (if yes – by whom?)
   e. How did you prepare yourself for birth?

3. **How did you experience childbirth?**
   a. Duration?
   b. Mode of delivery (vaginal / cesarean)?
   c. What was important during childbirth?
   d. What was helpful during childbirth?
   e. Who was present?
   f. Were you needs sufficiently responded to by present persons?
   g. Was there something bothering?
   h. What happened immediately after childbirth / did your child remain with you?

4. **How did you experience the first couple of weeks after childbirth?**
   a. How did you experience first time after birth?
   b. Support after childbirth?
   c. Duration of hospital stay?
   d. Support appropriate and adequate?
   e. Support in care for newborn baby?
   f. Support in restructuring everyday life with the baby?

5. **In what manner could care for women with disabilities be optimized?**
   a. Structural conditions and equipment
   b. Structural processes
   c. Care and support
